# Comparative transcriptome and microbial community sequencing provide insight into yellow-leaf phenotype of *Camellia japonica*

**DOI:** 10.1186/s12870-021-03198-w

**Published:** 2021-09-10

**Authors:** Mingyue Fu, Zhongcheng Zhou, Xu Yang, Zhongbing Liu, Jiarui Zheng, Xinru Huang, Ling Wang, Jiabao Ye, Weiwei Zhang, Yongling Liao, Feng Xu

**Affiliations:** 1 College of Horticulture and Gardening, Yangtze University, 434025 Jingzhou, Hubei China; 2 Department of Forestry Ecology, Hubei Ecology Polytechnic College, 430070 Wuhan, China; 3 School of Horticulture and Landscape, Wuhan University of Bioengineering, 430415 Wuhan, China

**Keywords:** *Camellia japonica*, Leaf color variation, Chloroplast, Transcriptome, Microbial diversity

## Abstract

**Background:**

Leaf color variation is a common trait in plants and widely distributed in many plants. In this study, a leaf color mutation in *Camellia japonica* (cultivar named as Maguxianzi, M) was used as material, and the mechanism of leaf color variation was revealed by physiological, cytological, transcriptome and microbiome analyses.

**Results:**

The yellowing *C. japonica* (M) exhibits lower pigment content than its parent (cultivar named as Huafurong, H), especially chlorophyll (Chl) and carotenoid, and leaves of M have weaker photosynthesis. Subsequently, the results of transmission electron microscopy(TEM) exhibited that M chloroplast was accompanied by broken thylakoid membrane, degraded thylakoid grana, and filled with many vesicles. Furthermore, comparative transcriptome sequencing identified 3,298 differentially expressed genes (DEGs). KEGG annotation analysis results showed that 69 significantly enriched DEGs were involved in Chl biosynthesis, carotenoid biosynthesis, photosynthesis, and plant-pathogen interaction. On this basis, we sequenced the microbial diversity of the H and M leaves. The sequencing results suggested that the abundance of *Didymella* in the M leaves was significantly higher than that in the H leaves, which meant that M leaves might be infected by *Didymella*.

**Conclusions:**

Therefore, we speculated that *Didymella* infected M leaves while reduced Chl and carotenoid content by damaging chloroplast structures, and altered the intensity of photosynthesis, thereby causing the leaf yellowing phenomenon of *C. japonica* (M). This research will provide new insights into the leaf color variation mechanism and lay a theoretical foundation for plant breeding and molecular markers.

**Supplementary Information:**

The online version contains supplementary material available at 10.1186/s12870-021-03198-w.

## Background

Camellia (*Camellia japonica* L.) is an evergreen shrub of the genus *Camellia* and is widely distributed worldwide. *C. japonica*, as a woody oil plant, is primarily used as raw material for edible oil processing [1]. Camellia extract is widely used in medicine and is often used as an antioxidant [2], antitumor [3], antibacterial [4], and antiviral drug [5, 6]. It maintains a normal cholesterol level and protects the liver [7]. The camellia flower is one of the top 10 national flowers in China, and thus, camellia tree is often used in landscaping [8]. As an important ornamental species, *C*. *japonica* has a long history of artificial domestication and is often used as an outdoor shrub and a family-potted plant. At present, 32,000 cultivated species of camellia are reported worldwide [8, 9]. In general, the ornamental value of camellia is primarily attributed to its unique petal structures (simple, semidouble, and double flowers) [10, 11]. In addition, the leaves of camellia varieties are also used for ornamental purposes.

In higher plants, green leaves are primarily caused by the large accumulation of chlorophyll (Chl). Chl synthesis in higher plants is controlled by several genes, and the mutations of these genes result in various leaf color mutants [12]. For example, the undeveloped thylakoid membranes and yellow leaf phenotype of peas were caused by virus-induced silencing of *CHLI* and *CHLD* genes [13]. Over the past few decades, leaf yellowing mutants, such as *Cucumis sativus* [14, 15], maize [16], rice [17], wheat [18, 19], *Camellia sinensis* [20], *Brassica rapa* [21], and *Arabidopsis thaliana* [22, 23], have been identified in many plants. These mutants have been widely used in basic research. For example, Chl mutants have been used in analyzing gene functions, to reveal the development mechanism and photosynthetic characteristics of chloroplasts and the signaling pathway between the chloroplasts and nuclei [24, 25].

**Fig. 1:**
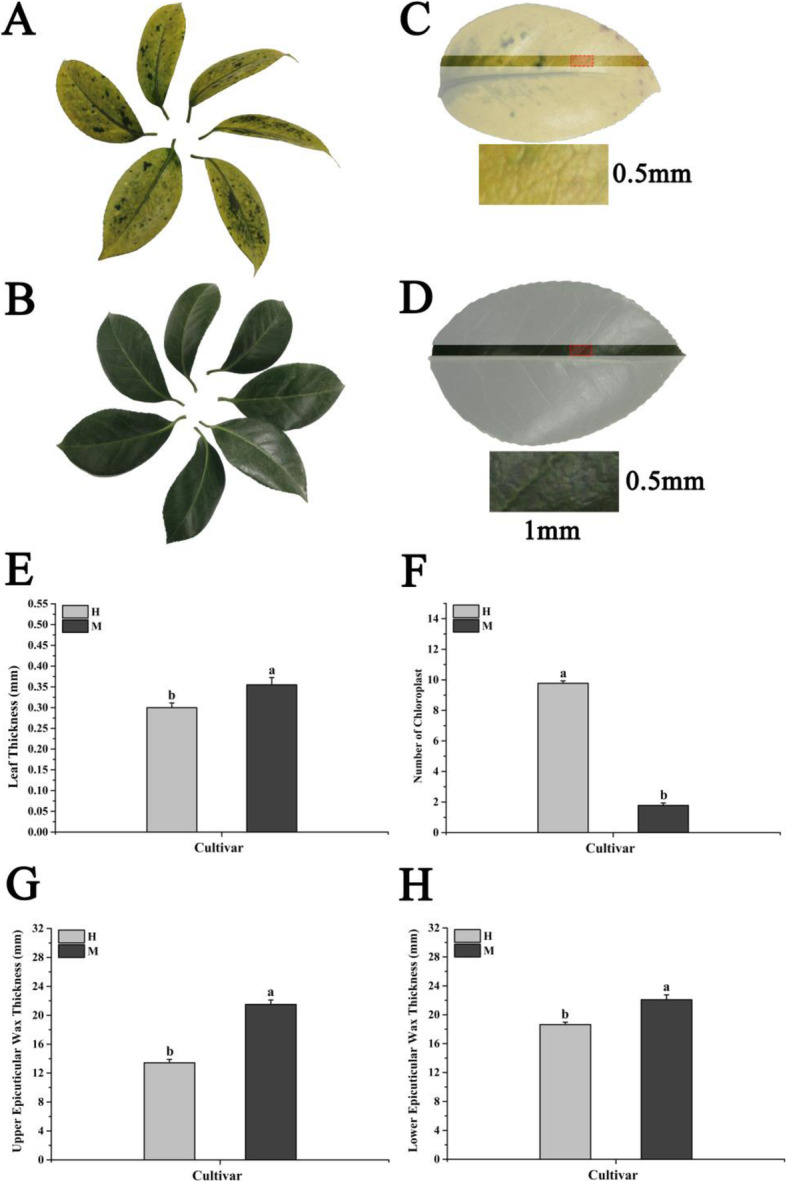
Phenotypes and microstructure differences between H leaves and M leaves. **A** **B** Phenotype of the leaf in H、M; **C** **D** Material of transmission electron microscope, the red-dashed box represents the sampling areas and the black font indicates the size of the material; **E** Comparison of average leaf thickness of each leaf in M and H; **F** Comparison of average number of chloroplasts per cell in the H leaves and M leaves; **G** Comparison of average upper epicuticular layer thickness of each leaf in M leaves and H leaves; **H** Comparison of average lower epicuticular layer thickness of each leaf in M leaves and H leaves. All data were shown as mean ± SE (*n* = 3). Means with different letters at each treatment represented a significant difference at *p* ≤ 0.05

With the development of breeding technology, green trees are far from meeting the needs of landscape engineering. Color-leafed tree species, such as *Cercis Canadensis* with purple leaves [26] and *Lagerstroemia indica* with yellow leaves [27], are widely used in gardens to improve the ornamental effect of gardens. Germplasm resources with the colorful leaves of tree species actually remain deficient; therefore, their breeding and exploitation are an urgent problem to solve. As one of the main sources of color-leafed trees, leaf color variation is critical for improving landscape ecological construction. For example, the cultivar ‘Royalty’ of *Malus* crabapples has remarkable purple leaves, which is different from many varieties of ornamental crabapple. Thus, *M*. ‘Royalty’ is frequently used as parent materials to breed color-leafed descendants [28]. At the early stage of the present study, a new *C. japonica* cultivar ‘Maguxianzi’ (M, the cultivar certificate is shown in Additional file 1: Figure S1) with yellow leaf was selected breeding from the parent *C. japonica* cultivar Hafurong (H). The leaves of M are mottled golden or even yellow and have high ornamental value, which will be expected to become a new color-leafed garden tree (Fig. 1A). Given that this cultivar has a visible trait, M is propitious for street greening, courtyard adorning, park landscaping, and other attractions. Predictably, M could possibly be extended as a scion for *C. japonica* varieties of interest for fresh markets. Moreover, the application of M, as a novelty cultivar of *C. japonica*, will add competitiveness to the sector and considerably increase the profit margin.

In this study, two cultivars of *C. japonica* (H and M) were selected as the test materials by comparing the microstructures and ultrastructures of chloroplasts in the leaves of the two cultivars and measuring physiological indices, such as chloroplast pigment content, photosynthesis parameters, and Chl fluorescence parameters. Furthermore, the differentially expressed genes (DEGs) of H and M were identified through comparative transcriptome analysis. On this basis, the microbial species and their abundances were compared by microbial diversity sequencing, and the candidate microorganisms infecting the *C. japonica* leaves and causing the yellow leaf phenotype are screened. This research result provides a theoretical basis for elucidating the physiological and molecular variation mechanisms of leaf color in *C. japonica*.

## Results

### Physiological changes in the yellow leaves

We compared the differences in photosynthesis parameters, chlorophyll fluorescence parameters, Chl, and carotenoid content between the M and H leaves to explore the differences in physiological and biochemical indices between H and M leaves. The results show that the net photosynthetic rate (Pn), stomatal conductance (Gs), intercellular CO_2_ concentration (Ci), transpiration rate (Tr), and water use efficiency (WuE) of M are significantly lower than those of H (Table 1). The comparative analysis of chlorophyll fluorescence parameters between the two cultivars shows that the maximum photochemical efficiency (Fv/Fm) of the M leaves is significantly lower than that of the H leaves (Table 1). Chl a, Chl b, total Chl, and carotenoid contents in the M leaves are significantly lower than those in the H leaves (Table 1).

**Table 1:** Determination of physiological indexes in leaves

Physiological indexes		H	M
Photosynthesis parameters	Pn (µmol·m^− 2^·s^− 1^)	5.3413 ± 0.9168 a	3.2563 ± 0.9505 b
	Gs (mol·m^− 2^·s^− 1^)	0.6167 ± 0.0025 a	0.3333 ± 0.0041 b
	Ci (µmol·mol^− 1^)	273.54 ± 5.4665 a	220.72 ± 5.9231 b
	Tr (mmol·m^− 2^·s^− 1^)	1.0480 ± 0.1209 a	0.752 ± 0.0325 b
	wuE (mmol·mol^− 1^)	5.4775 ± 0.1802 a	4.3379 ± 0.2823 b
Chlorophyll fluorescence parameters	Fv/Fm	0.6390 ± 0.0147 a	0.5283 ± 0.0182 b
Pigments	Chl a (mg·g^− 1^)	1.3383 ± 0.1007 a	0.1527 ± 0.0498 b
	Chl b (mg·g^− 1^)	0.5222 ± 0.0812 a	0.0534 ± 0.0034 b
	Carotenoid (mg·g^− 1^)	0.2488 ± 0.0279 a	0.0666 ± 0.0012 b

The units of each parameter are shown in parentheses. Data were analyzed by SAS, followed by Duncan’s honestly significant difference test at *p* ≤ 0.05. All data shown reflect the average mean of three biological replicates (*n* = 3)

### Cytological changes in the yellow leaves

**Fig. 2:**
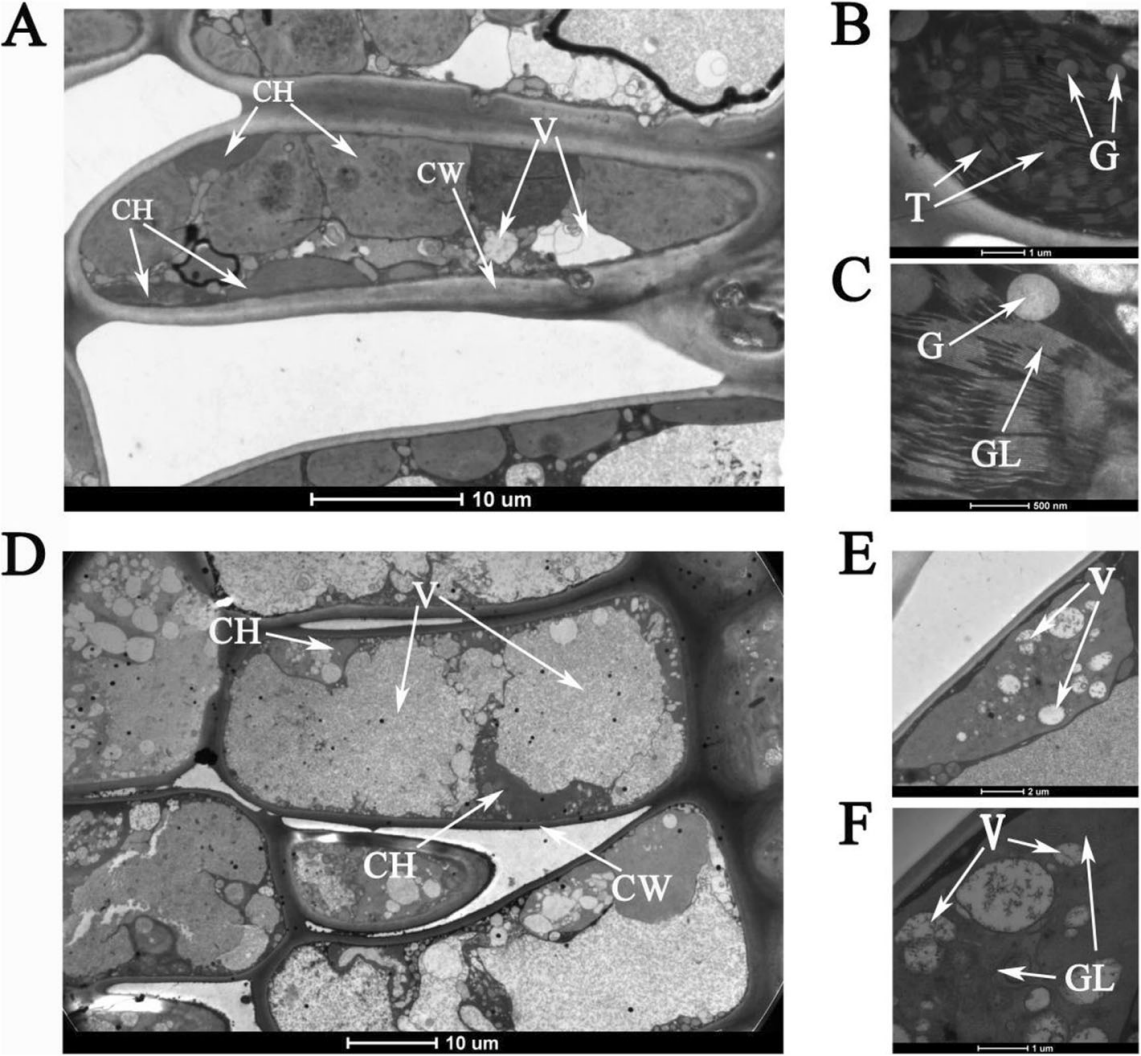
Ultrastructure analysis of chloroplast between H leaves and M leaves. **A** **B** **C** The ultrastructures suggest the typical structures of chloroplast and thylakoid in H leaves. **D** **E** **F** The ultrastructures suggest destroyed structures of chloroplast, degraded thylakoid, and filled with various sized vesicles in the chloroplast stroma in M leaves. **E** **F** Traces of abnormal degradation of thylakoids in abnormal chloroplasts. CH chloroplast, CW cell wall, V vacuole, T thylakoid grana, G granulose, and GL grana lamella

The comparative analysis of the microstructures shows that the thicknesses of the wax layer and upper/lower epidermis of the M leaves are significantly higher than those in the H leaves (Fig. 1E, G, H). The ultrastructure analysis results show that the chloroplasts in the mesophyll cells of the H leaves present normal structures, and they contain small starch granules, typical thylakoid membrane, and stromal lamellae (Fig. 2E, F). By contrast, in the M leaves, the thylakoid membranes of the chloroplasts are broken and have no typical or lack thylakoid grana and have several irregularly arranged vesicles (Fig. 2B, C). In the M leaves, many vacuoles are present in the chloroplasts, which indicates the vacuolation of the chloroplast and degradation of the chloroplast structures (Fig. 2A, B). The sizes and number of chloroplasts in the cells of the M leaves are significantly lower than those in the cells of the H leaves (Figs. 1 F and 2D, E).

### Overview of transcriptome sequencing

According to the changes in phenotype, ultrastructure, and physiology mentioned above, the expression patterns of the genes involved in chloroplast development and pigment biosynthesis in the yellow leaves display putative changes. Therefore, comparative transcriptome analysis was conducted on the M and H leaves in this study. Six cDNA libraries (H1, H2, H3, M1, M2, M3) were sequenced by Illumina HiSeq 2500 platform, and 286.49 million raw reads and 42.97 Gb of raw bases were generated. After data filtering, 274.29 million clean reads and 41.14 Gb of clean bases were obtained. The proportion of the clean reads in all libraries exceeds 94 % of the total reads (Additional file 2: Table S1), and the clean reads were stored in NCBI sequence reading archive (SRA accession number: PRJNA577010). Q30 in these libraries exceeds 94 %, and the GC content is in the range of 45.44-47.42 % (Additional file 2: Table S1). HISAT v2.0.4 software was used to map the clean reads to the reference gene database of the *C. sinensis* [29]. The matching rates of the cDNA libraries of the samples range from 65.49 to 69.24 % (Additional file 3: Table S2). More than 53 % of reads in these samples can be compared with the exon region of the reference genome (Additional file 4: Table S3). The correlation analysis of the expression levels of various samples shows good repeatability (Additional file 5: Figure S2). Overall, the quality of the high-throughput sequencing data by Illumina is sufficiently high to satisfy the requirements of the transcriptome data. Thus, these data can be used for further DEG analysis.

### Functional annotation and classification of DEGs

A total of 3,298 DEGs were obtained from the H and M leaves. Among these DEGs, 1,812 DEGs are upregulated, and 1,486 DEGs are downregulated (Additional file 6: Figure S3 and Additional file 7: Figure S4). According to the expression of DEGs in each sample, K-means cluster analysis shows that DEGs are divided into six clusters (Additional file 8: Figure S5).

**Fig. 3:**
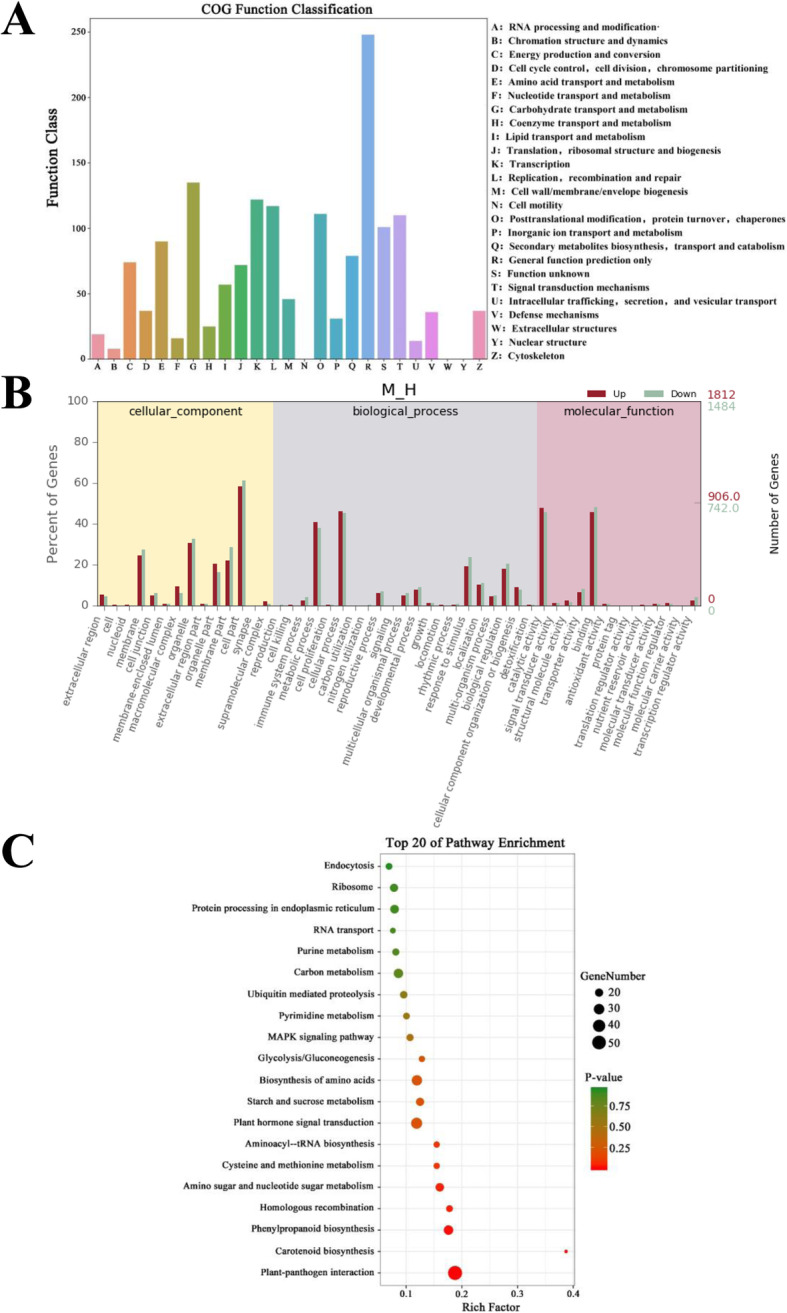
Function annotations of the DEGs in the leaves of the *C. japonica*. **A** COG annotation of the DEGs in the leaves. **B** GO annotation of the DEGs in the leaves. **C** KEGG annotation of the DEGs in the leaves

COG, GO, and KEGG annotation was conducted for the prediction of the functions and classification of all DEGs. A total of 3,298 DEGs were annotated functionally and classified in 24 COG categories, including cell structure, biochemical metabolism, signal transduction, and so on (Fig. 3A). Among these categories, the group of general function prediction contains the largest number of DEGs (245, 7.43 %), followed by carbohydrate transport (137, 4.15%); transcription (119, 3.61%); replication, recombination and repair (113, 3.43%) as well as posttranslational modification, protein turnover, chaperones (108, 3.27%).

GO annotation result indicates that 3,298 DEGs are primarily assigned to 48 function items (Fig. 3B). In cellular components, DEGs are primarily concentrated in the cell part (1,968, 59.67 %), organelle (1,035, 31.38%), and membrane (848, 25.71%). In biological processes, most of the DEGs are involved in cellular process (1,509, 45.76%), metabolic process (1,305, 39.57%), and stress to stimulus (698, 21.16%). In molecular function, most DEGs are concentrated in binding (1,545, 46.85%) and catalytic activities (1,544, 46.82%).

KEGG annotation reveals that 3,298 DEGs are enriched in 123 KEGG pathways. In these pathways, DEGs are most significantly enriched in plant-pathogen interaction and carotenoid biosynthesis pathways (Fig. 3C). According to the KEGG annotation result, we selected five KEGG pathways with significantly enriched DEG for subsequent analysis, namely, porphyrin and Chl metabolism (map00860), carotenoid biosynthesis (map00906), photosynthesis-antenna proteins (map00196), photosynthesis (map00195) and plant-pathogen interaction (map04626).

### DEGs related to pigment synthesis in the leaves

**Fig. 4:**
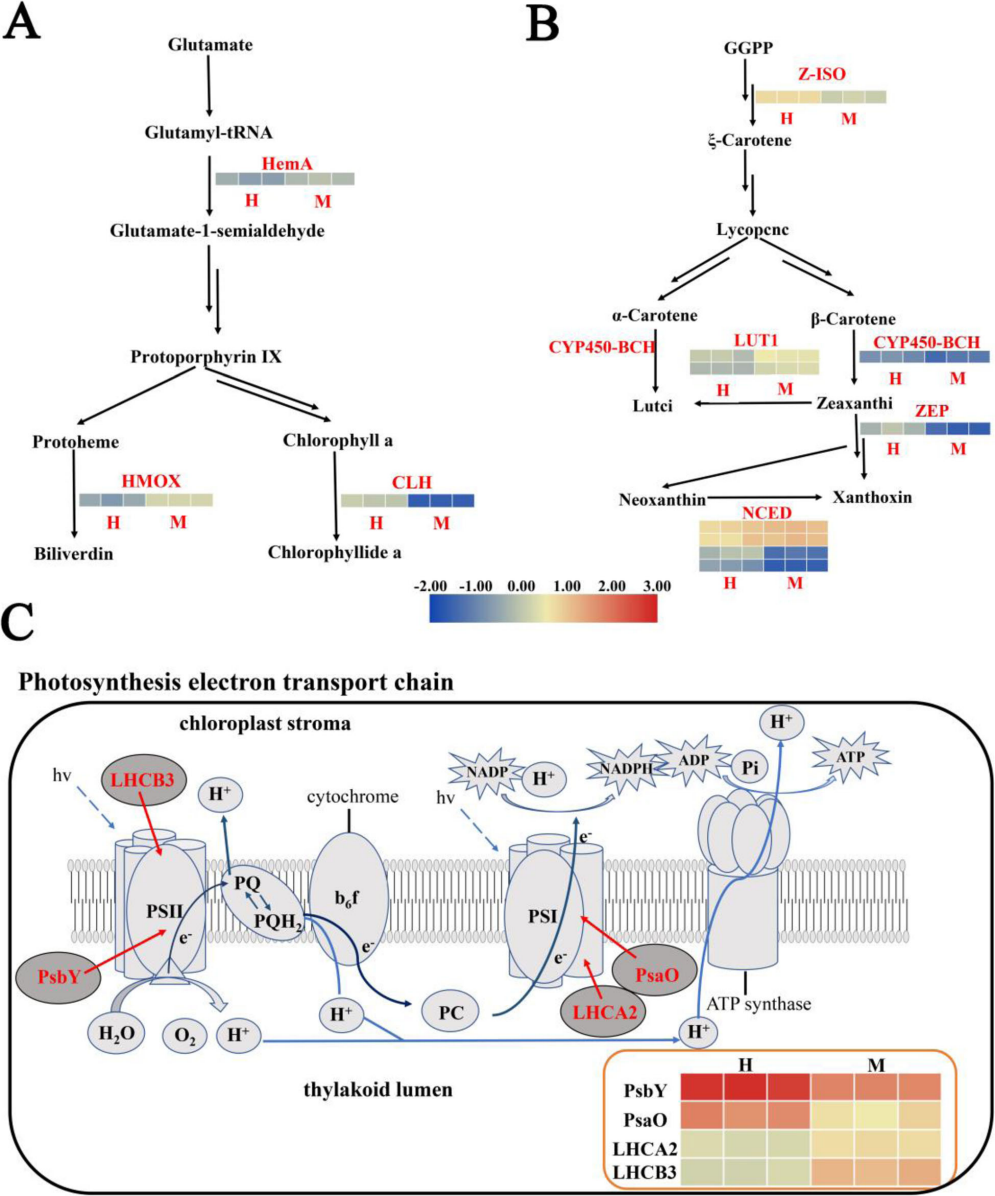
Pathway related to pigment synthesis in the leaves of *C. japonica* and expression profile of DEGs. **A** Chl metabolism route and the expression trend of DEGs related to this pathway in different samples. **B** Expression profiles of DEGs related to the carotenoid biosynthesis pathway in different samples. **C** Photosynthesis pathway and the expression trend of DEGs related to this pathway in the leaves of H and M. The expression trend of DEGs in different samples indicated by a color change in square, and the color change from blue to yellow suggests an increase in expression level of genes

In the transcriptome data, we analyzed the DEGs in the porphyrin and Chl metabolism pathways, which controls Chl biosynthesis. KEGG annotation results show that three DEGs are involved in Chl synthesis (Fig. 4A). Among these DEGs, the expression levels of the *glutamyl-tRNA reductase* (*HemA*) and *heme oxygenase* (*HMOX*) significantly increase, whereas the expression of *chlorophyllase* (*CLH*) decreases significantly in M compared with that in H.

In the M leaves, 12 DEGs are significantly enriched in the carotenoid biosynthesis pathway. Five of these DEGs are downregulated, namely, *Z-ISO, CYP450-BCH, ZEP, NCED3*, and *NCED4* (Fig. 4B). This finding is consistent with the result that carotenoid content in the M leaves is significantly lower than that in the H leaves.

### DEGs related to photosynthesis in the leaves

In the photosynthesis-antenna protein pathway, the expression levels of two DEGs (*LHCA2* and *LHCB3*) in the M leaves are significantly higher than those in the H leaves. By contrast, the expression levels of two DEGs (*PsaO* and *PsbY*) are significantly downregulated in the M leaves compared with those in the H leaves in the photosynthetic pathway (Fig. 4C).

### DEGs related to plant-pathogen interaction in the leaves

**Fig. 5:**
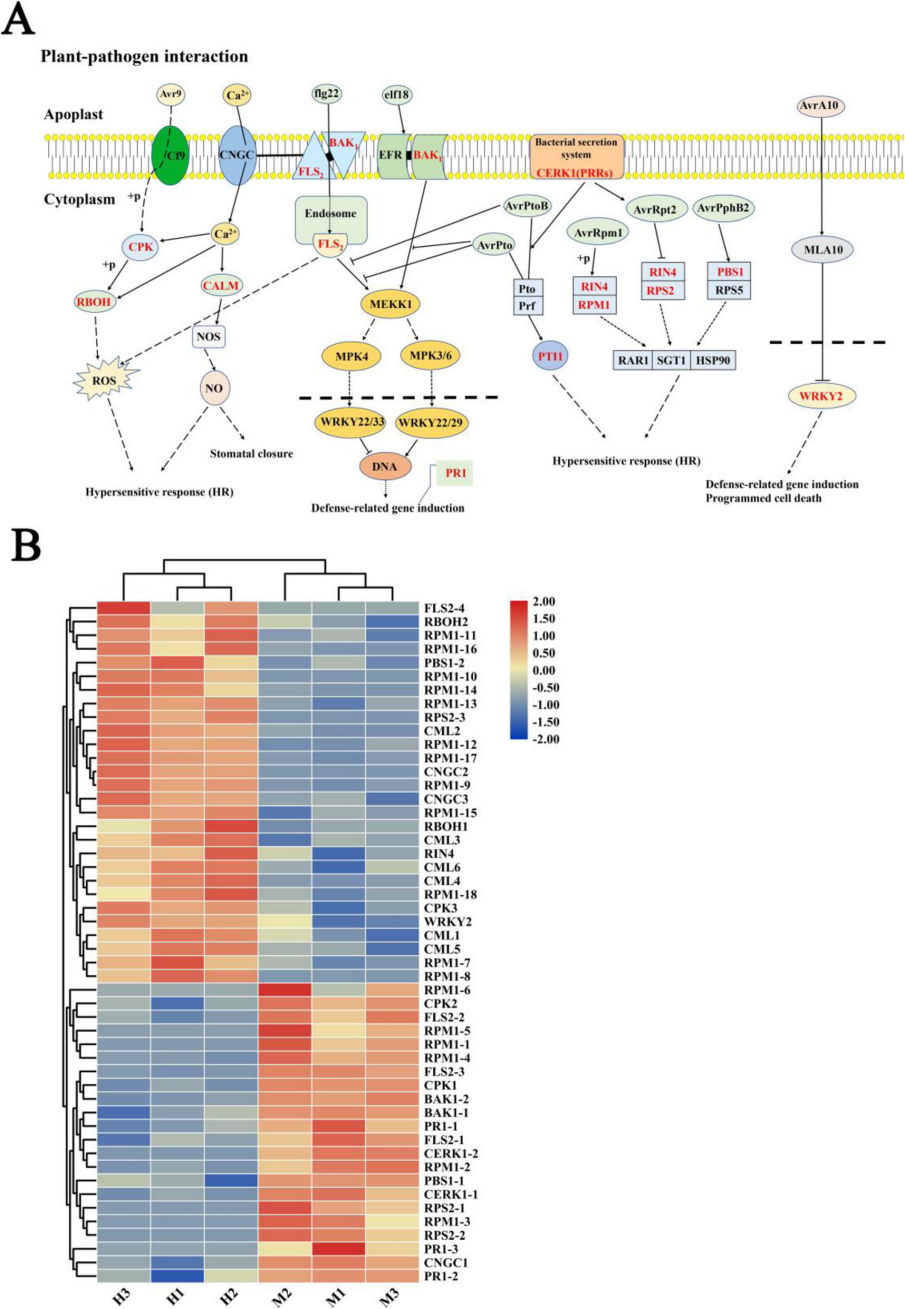
Plant-pathogen interaction pathway in two samples and expression profiles of DEGs. **A** Schematic diagram of the plant-pathogen interaction pathway in the leaves of H and M [30], DEGs are marked in red. **B** The expression profiles of DEGs of plant-pathogen interaction pathway in the leaves of *C. japonica*. The expression trend of DEGs in different samples indicated by a color change in square, and the color change from blue to yellow suggests an increase in expression level of genes

KEGG annotation result shows that 50 DEGs are enriched in the plant-pathogen interaction pathway (Figs. 5A and 3B). Among these DEGs, 19 are upregulated in the M leaves, primarily including *RPM1*, *RIN4*, *CPK*, *BAK1*, *PTI1* and *PR1*, and 31 are downregulated. The further function analysis of 50 DEGs shows that the expression levels of the specific recognition receptors located on the cell membranes (i.e., *BAK1* and *FLS2*) in the M leaves significantly increase compared with those in the H leaves. Additionally, the expression level of the plant resistance gene *PR1* in M is significantly upregulated (Fig. 5A and B).

### Richness and diversity of microbial community in the leaves

**Fig. 6:**
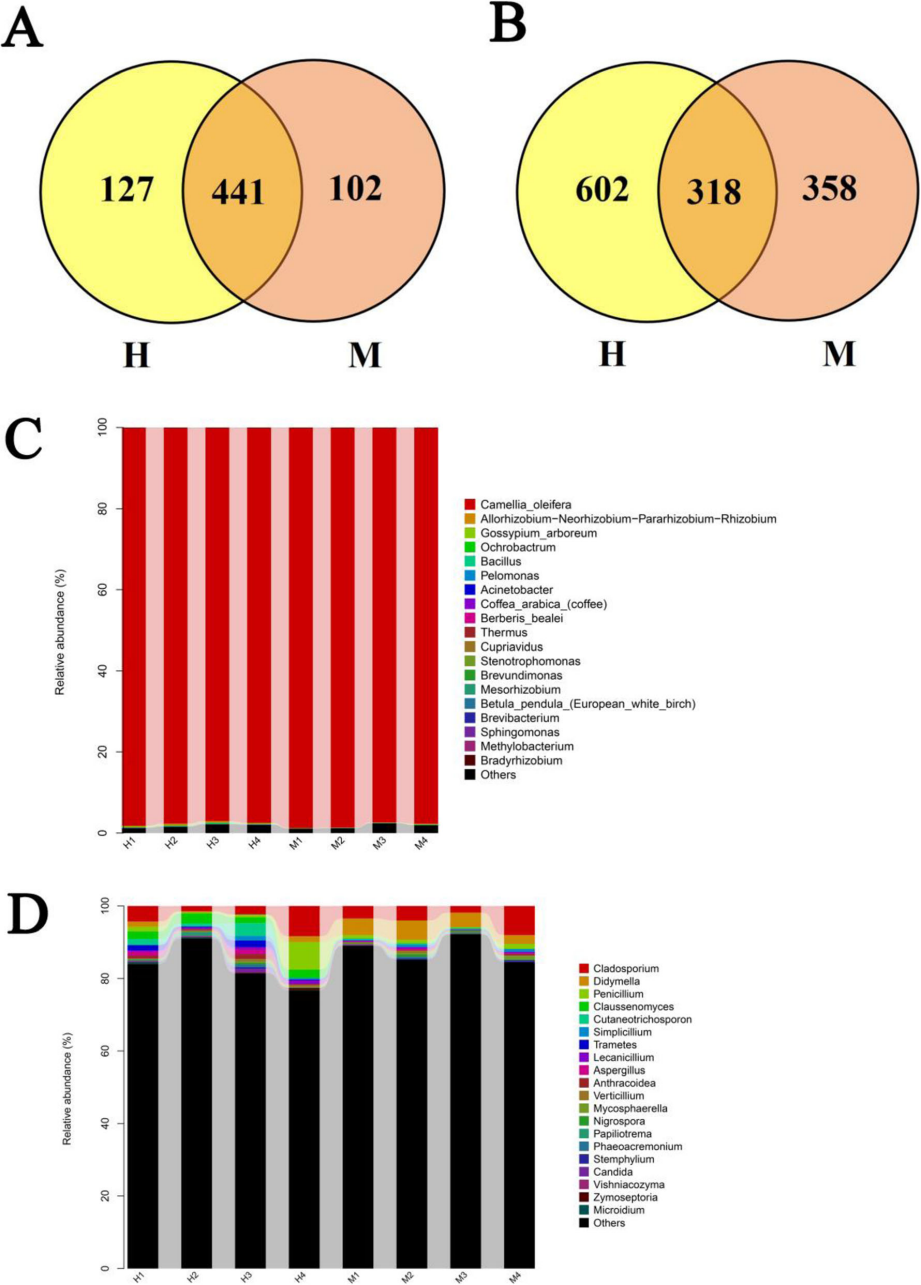
Composition of microbial diversity in each group. **A **Venn diagram of shared OTUs in prokaryote. **B** Venn diagram of shared OTUs in eukaryote. Relative abundance of prokaryotic **C** and eukaryotic **D** communities in different treatment at genus level

Transcription annotation results show the highest significant difference in DEG in the plant-pathogen interaction pathway. Therefore, the yellow leaf of M may be caused by the endogenous microorganisms in the leaves. Thus, we sequenced the 16 S and ITS diversity of the leaves of both groups, compared the species and abundances of prokaryotic and eukaryotic microorganisms in H and M leaves, and screened the candidate microorganisms involved in M leaf color variation. The sequencing result shows that 224,313 and 214,939 effective sequence reads are obtained by 16 S rRNA and ITS diversity sequencing, respectively. In prokaryotes, the total number of OTUs is 670, and 441 shared OTUs can be detected in the two groups (Fig. 6A). In eukaryotes, the total number of detected OTUs is 1,278, of which 318 OTUs are shared by the two groups (Fig. 6B).

**Fig. 7:**
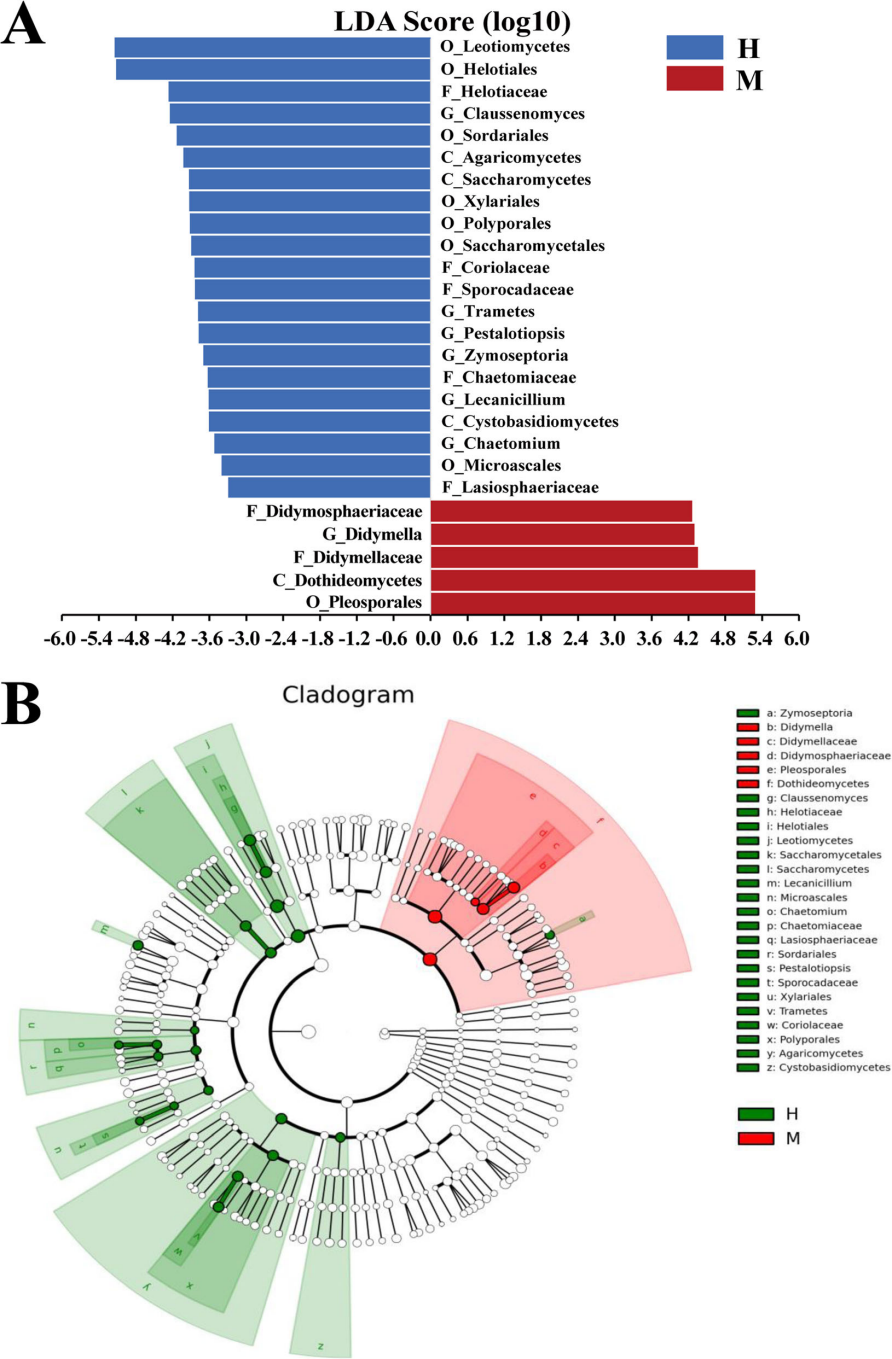
Comparative analysis of fungal community abundance between H samples and M samples. **A** LEfSe difference analysis of the relative abundance of fungal communities between H leaves and M leaves. The length of the column indicates the significant degree of the difference in the taxa, and the different colors of the bar graph indicate the higher abundance sample groups corresponding to the taxa. C: Class; O: Order; F: Family; G: Genus. **B** Diagram of the classification unit based on the classification hierarchical tree. The classification hierarchy tree shows the hierarchical relationship of all taxa from the phylum to the genus (arranged from the inner circle to the outer circle) in the sample fungal community. The size of the node corresponds to the average relative abundance of the taxa. Green indicates that the taxa has a higher abundance in H, while red indicates that the taxa has a higher abundance in M. The names of all taxa with significant differences are listed on the right

For prokaryotes, the differences in community classification and abundance between the M and H leaves are nonsignificant (Fig. 6C). However, for eukaryotes, the classification result shows that OTUs belonging to 159 genera under eight phyla are found. Further comparative analysis on the abundance of these eukaryotes reveals that the average abundance of *Didymella* in the M leaves (2.4-5.2 %) is significantly higher than that in the H leaves (0.0-2.4 %) (Fig. 6D). The LEfSe difference analysis of the relative abundance of fungal communities between the two groups of samples through the Galaxy online analysis platform (http://huttenhower.sph.harvard.edu/galaxy/). The results showed that the abundance of the fungal communities of taxa such as *Dothideomycetes*, *Pleosporales*, *Didymellaceae*, and *Didymella* are higher in M than in H (Fig. 7A). Furthermore, a diagram of the classification unit based on the classification hierarchical tree is constructed to find candidate fungal caused yellow leaf, and the results indicated that the abundance of fungal community in the family, order, and class where *Didymella* belongs was higher in M than that in H. The above results showed that *Didymella* may be closely related to the yellow leaf of *C. japonica* (Fig. 7B).

### Validation DEGs by qRT-PCR

**Fig. 8:**
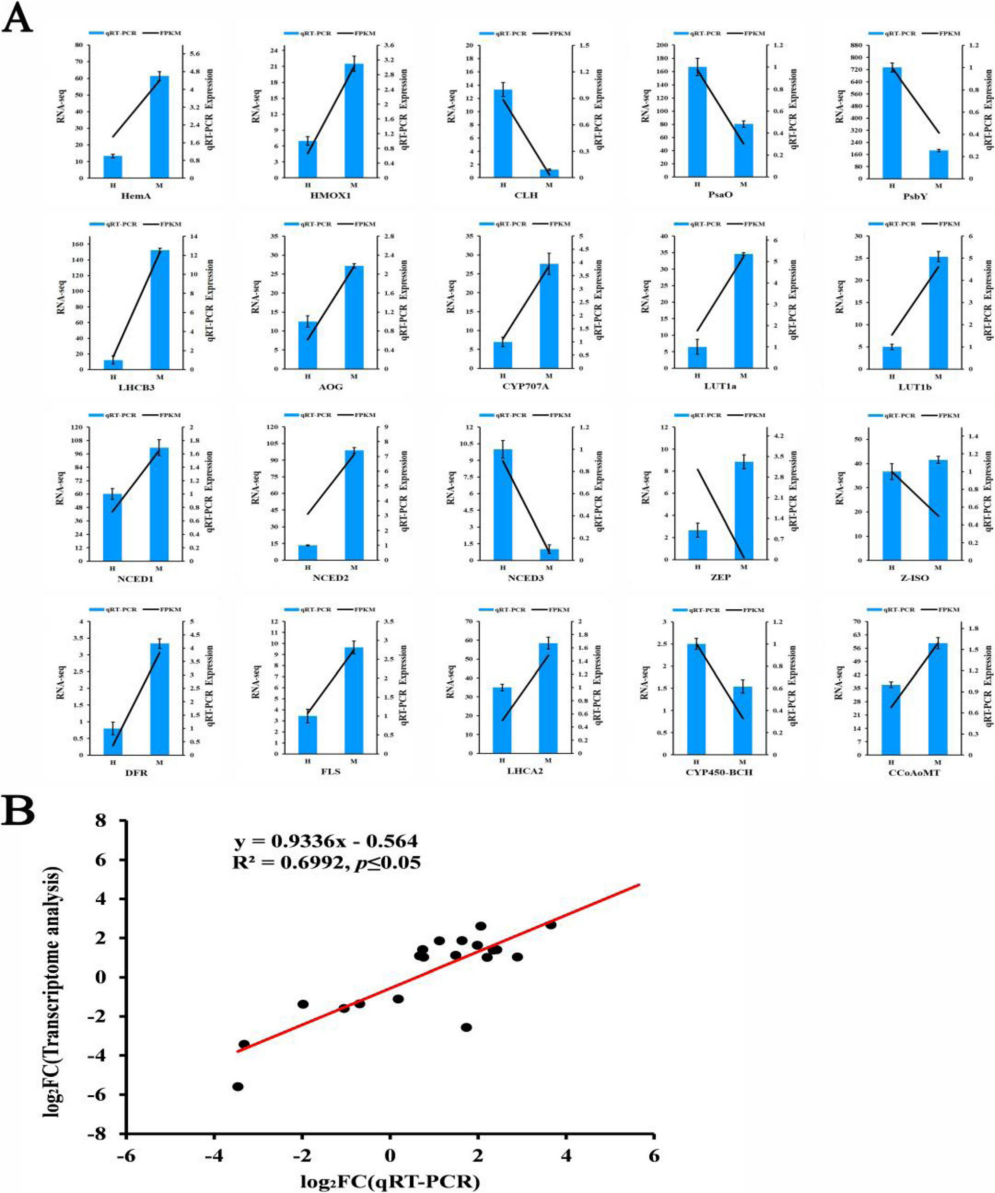
qRT-PCR verification diagram of DEGs in the M leaves and H leaves. **A** Result of expression level compared between qRT-PCR and RNA-seq data in two sets of sample leaves. **B** Correlation analysis between the FPKM and qRT-PCR results. The result was obtained using log_2_(foldchange) measurements. R^2^ value represents the strength of correlation between RNA-seq and qRT-PCR results. All data are shown as mean ± SE (*n* = 3). Means with different letters at each treatment represent a significant difference at *p* ≤ 0.05

We verified the expression of 20 DEGs that were randomly selected in the two groups of samples by qRT-PCR to confirm the reliability of the RNA-seq data (Fig. 8A). Log_2_foldchange was used to calculate the correlation between the RNA-Seq and qRT-PCR results (2^−ΔΔCt^) of 20 DEGs. The result shows that the qRT-PCR result of the 20 DEGs is positively correlated to the RNA-seq result, which means the RNA-seq data are reliable and accurate (Fig. 8B).

## Discussion

### Obstructed chlorophyll synthesis as an important factor for the yellowing of M leaves

**Fig. 9:**
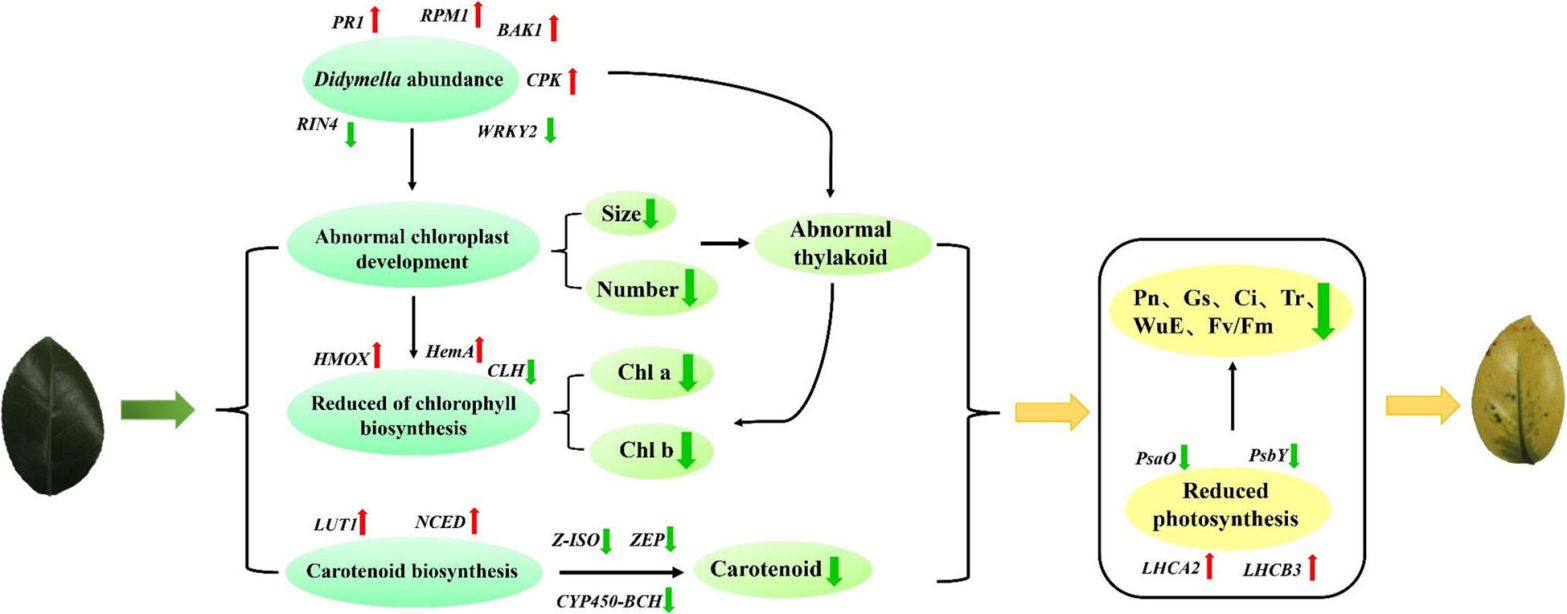
Regulation model of yellow leaf variation in *C. japonica*. Leaf color variation is mainly associated with elevated *Didymella* abundance, abnormal chloroplast development, reduced Chl biosynthesis, reduced carotenoid biosynthesis, and reduced photosynthesis. Key DEGs in this process are indicated in italics font. A red upward arrow represents a rise and a green downward arrow represents a decrease or decline

Leaf color is an important commercial feature of ornamental plants. The color of a plant leaf primarily depends on the content and distribution of Chl, carotenoid, and anthocyanin [31]. In general, green leaves are primarily caused by high Chl concentration, whereas colorful leaves are primarily caused by changes in carotenoid and anthocyanin contents. The M shows yellow leaf phenotype varied from the green leaf of H. The measured pigment content indicates that Chl content in the M leaves is significantly decreased. Transcriptome KEGG annotation result shows that three DEGs, namely, *HemA*, *HMOX* and *CLH*, are involved in Chl biosynthesis (Fig. 9). In contrast to the decline in Chl content, the two key genes, namely, *HemA* and *HMOX*, which control the Chl biosynthesis, are significantly upregulated in M compared with those in H [32]. Moreover, the *CLH* gene plays an important role in the degradation and metabolism of Chl, and its expression level in the M leaves is lower than that in H [33].

In nature, leaf color is the result of plant evolution. The leaf lacking Chl may lead to decreased biomass, fecundity, and adaptability to adversity of plants [34, 35]. Thus, plants have a series of feedback regulation mechanisms to weaken or eliminate this influence [36]. The expression levels of the three DEGs involved in Chl synthesis in the M and H leaves are the opposite to the change in Chl content. This result may be due to the change in expression level. This change is further mediated by the cell nuclei that receive the reverse signals of plastids transferring chloroplast development and functional status after the M leaves show Chl deficiency. As Chl is an important photosynthetic pigment, Chl deficiency in the M leaves results in decreased Pn. Meanwhile, the expression levels of DEGs in the photosynthesis pathway change accordingly.

Carotenoids comprise a series of pigments from yellow to red, which are involved in light capture in photosynthesis and are essential to the protection of plants exposed to excessive light [37, 38]. Therefore, impaired carotenoid biosynthesis can lead to a color mutant phenotype [39, 40]. Leaf color mutation in many plants is related to the synthesis and degradation of carotenoids [41, 42]. In the present study, DEGs are significantly enriched in the carotenoid biosynthesis pathway. The qRT-PCR result shows that five DEGs, namely, *Z-ISO*, *CYP450-BCH*, *ZEP*, *NCED3*, and *NCED4* are downregulated in the M leaves, and the rate of carotenoid biosynthesis in the M leaves decreases significantly (Fig. 9). Finally, the M leaves turn yellow.

### Destruction of chloroplast structure leads to yellowing of the M leaves

The development of chloroplast is the main affecting factor of the formation of leaf color in higher plants. Various leaf color mutants caused by chloroplast dysplasia have been reported in many species, such as *L. indica* [27], tea tree [43], and *Ginkgo biloba* [44, 45]. In general, the leaves in mutants with abnormal chloroplast function show Chl deficiency. The number and development of abnormal chloroplasts may lead to impaired thylakoid membranes and reduced accumulation of light trapping proteins in photosystems I and II [13]. Therefore, change in leaf color may reflect the abnormal development and function of chloroplasts. In view of the significantly decreased chloroplast pigment in the M leaves, the ultrastructures of the chloroplasts in the H and M leaves were explored through transmission electron microscopy. The result shows that the number of chloroplasts in the M leaves is significantly lower than that in the H leaves. The chloroplast structures are incomplete and replaced by many bubbles of different sizes, and dense thylakoid grana are nearly absent. Thylakoids play an important role in plant photosynthesis. Photosynthetic pigment synthesis and photosynthetic electron transfer occur around thylakoids and their surfaces [46]. Meanwhile, Chl is embedded in the thylakoid membrane of a chloroplast. The ultrastructure analysis result indicates that the significantly decreased Chl content in the M leaves is caused by the destruction of the chloroplast structures and results in yellow leaf phenotype.

### *Didymella* causes yellowing leaves in M by destroying the chloroplast structures

Comparative transcriptome analysis result suggests that the DEGs in the H and M leaves are significantly enriched in the plant-pathogen interaction pathway (50 DEGs). Furthermore, the expression levels of the pattern recognition receptor, LRR receptor-like serine/threonine-protein kinase (*FLS2*), and brassinosteroid insensitive 1-associated receptor kinase 1 (*BAK1*) are significantly upregulated in the M leaves (Fig. 9). These enzymes form a complex with bacterial flagella flg22 to activate plant defense signal as resistance to pathogen invasion [47, 48]. The expression levels of the *CML* and *CPK* genes encoding Ca^2+^ signal channel-related proteins or protein kinases in the M leaves are significantly increased compared with those in the H leaves. Ca^2+^ channel-related genes play key roles in plant resistance to biotic stress [49]. Changing Ca^2+^ concentrations in cell solutes is the main response of plant organisms to biotic and abiotic stresses [50]. The expression levels of Ca^2+^ channel-related genes significantly increase in the M leaves. These genes can activate respiratory burst oxidase, and mediate the production of reactive oxygen species, and they are involved in the immune response of plants [51, 52].

Plants control the activation of innate immune response through process involving mitogen-activated protein kinases and salicylic acid. These immune defenses include the deposition of lignin and callose in the cell wall, transcription of related disease-resistant genes, and production of antibacterial compounds and reactive oxygen species [53]. The wax layer and upper/lower epidermis in the M leaves are thicker than those in the H leaves. This result is due to the effect of lignin deposition in the epidermal cells of the M leaves. After the M leaves show a Chl-deficiency phenotype, the plant increases its resistance to pathogens by increasing the thickness of leaf epidermis and wax layer.

Many studies have reported that various plant pathogens promote pathogenesis by injecting their different type III effectors (T3E) into plant cells [54]. T3E, which is commonly found in *Pseudomonas syringae*, has a putative chloroplast targeting sequence and a J domain, which can activate 70 kDa heat shock protein and thus promote the remodeling of chloroplast thylakoid structures and inhibit of salicylic acid accumulation [55, 56]. In this study, the KEGG annotation result shows that the plant-pathogen interaction pathway is a DEG-enriched pathway with the most significant difference. At the same time, the results of microbial diversity sequencing show that the abundance of *Didymella* genus in M leaves is significantly higher than that in the H leaves. Therefore, the eukaryotes of genus *Didymella* is closely related to the yellow leaf phenotype of M. The *Didymella* may also have similar functions to *P. syringae*, as previously reported. It may specifically target the chloroplast of the plant cells and act on the thylakoid, thereby resulting in the degradation of the thylakoid grana and then causing the loss of Chl biosynthesis and yellow leaf phenotype in M (Fig. 9).

## Conclusions

In the yellow leaves of *C. japonica* (M), Chl and carotenoid content decreased, chloroplast ultrastructure was abnormal, the photosynthesis and photosynthetic product biosynthesis were affected. A total of 3,298 DEGs were identified by comparative transcriptome sequencing analysis. These DEGs are primarily involved in the plant–pathogen interaction, carotenoid biosynthesis, photosynthesis, and Chl biosynthesis. Moreover, microbial diversity analysis showed that the abundance of *Didymella* genus in M leaves is significantly higher than that in the H leaves. Overall, our data showed that the yellow-leaf phenotype of M is closed related to the decreased Chl content, destroyed chloroplast structure, and *Didymella* invasion. Conclusively, we speculate that *Didymella* in the M leaves may target chloroplasts and destroy the structures of thylakoids, and thus degrade thylakoid grana; this degradation reduces the Chl and carotenoid contents, and leads to yellow leaf phenotype of *C. japonica* (M).

## Methods

### Plant materials

The cultivars of *C. japonica* used in this study were planted in Wunao Mountain National Forest Park (31°13′44′′N, 114°59′17′′E) in Macheng, Hubei Province, China. All samples of *C. japonica* leaves were collected with the permission of the head of Wunao Mountain National Forest Park in Macheng. The certification of all *C. japonica* varieties is completed by the Camellia Branch of China Flower Association, and the certification of M is shown in Figure S1. In November 2017, leaves of similar sizes were collected from 15-year-old M and parent H plants with similar genetic backgrounds. The control was H, and the treatment was M (Fig. 1A − D). Three biological replicates were set for each cultivar. and 30 leaves were used in each round of transcriptome sequencing, microbial diversity sequencing, and physiological index determination. All samples were stored in -80 °C after quick freezing in liquid nitrogen.

### Measurements of Chl, carotenoid, photosynthesis parameters, and chlorophyll fluorescence parameters

The 15-year-old M and parent H plants with similar genetic backgrounds were selected, and the photosynthesis and chlorophyll fluorescence parameters of the leaves were determined with an LI-6400XT portable photosynthesis measurement system and portable chlorophyll fluorescence meter PAM-2500, respectively. Yellow leaves from M and green leaves H were selected, and Chl a, Chl b, and carotenoid contents were extracted and determined according to the method of Lichtenthaler et al. [57]. Each treatment was set up with three replicates, and 30 leaves from six trees were selected for each repetition.

### Transmission electron microscopy and optical microscopy observation

The leaves were first soaked in a mixture of 2.5 % glutaraldehyde and 2 % paraformaldehyde and then fixed with 1 % osmium tetroxide (Fig. 1C and D). Subsequently, the specimens were dehydrated, embedded, sectioned, stained, and observed [58]. For the observation of the microstructure, a 0.5 mm thick slice was cut with a Leica RM2265 semi-thin microtome, and leaf cells were observed and photographed with a Leica DVM6 digital microscope. Ten locations were randomly selected for the upper and lower epidermis of the leaves, and the thicknesses of the upper and lower epidermis and the waxy layer of the leaves under different treatments were measured with AutoCAD. For the observation of ultrastructures, the leaves were cut into 70 nm thick slices with a Leica EM UC7 ultra-thin slicing machine (Leica Microsystems GmbH, Wetzlar, Germany) and then stained with 3 % uranium acetate and 6 % lead citrate. Leaf cell structures and photographs were observed using Tecnai G2 Spirit Bio TWIN. Ultrastructural images were used in counting the chloroplasts in 10 randomly selected cells, and the average number of chloroplasts in each cell was calculated.

### RNA extraction and cDNA library preparation

Total RNA in *C. japonica* leaves was extracted according to the instructions of the TaKaRa MiniBEST Plant RNA extraction kit, and the purity and concentration of RNA were detected through agarose gel electrophoresis and with a NanoDrop 2000 microspectrophotometer (IMPLEN, CA, USA), respectively. The construction and sequencing of the cDNA library were completed by the Annoroad Gene Technology Corporation (Beijing, China). In this study, two sets of samples were constructed, and each set has three replicates and 30 leaves. Six cDNA libraries (H1, H2, H3, M1, M2, M3) were constructed, and each has 30 leaves. These cDNA libraries were sequenced using the Illumina HiSeq 2500 sequencing platform.

### Illumina deep sequencing and data analysis

Clean reads filtered from the raw reads were mapped to the tea tree reference genome database (Tea plant Genome Database http://www.plantkingdomgdb.com/tea_tree/) by using HISAT v2.0.4 [29]. All raw reads data were stored in the NCBI sequence read archive (SRA accession number: PRJNA577010) and transcriptome sequences were annotated with Trinotate. Functional annotations were mainly performed using databases, including PFAM, Nr, Swissprot, GO, COG, and KEGG.

### Identification and functional analysis of DEGs

The FPKM of RNA-Seq was used in estimating gene expression abundance [59]. The DESeq242 package [60] was used to identify DEGs in M and H, which used for GO and KEGG enrichment analysis [61, 62]. DEG enriched in the KEGG pathway was calculated using KOBAS [63].

### qRT-PCR analysis

DEGs were selected from the transcriptome for qRT-PCR analysis, and specific primers were designed using Primer 5.0 (Additional file 9: Table S4). The 18 S was selected as the internal reference gene [64], and qRT-PCR of DEG was performed by LineGene 9600 Plus real-time fluorescent PCR instrument (Bio-er, Hangzhou) and SYBR Green BioEasy Master Mix kit [65]. All reactions were performed in three biological replicates (30 leaves each repetition), and the relative expression levels of genes were calculated through the 2^−ΔΔCt^ method [66].

### Microbial diversity sequencing and analysis in leaves

Total genomic DNA was extracted using the Omega mag-bind Soil DNA kit (Noraville, GA, USA), and the concentration and quality of the extracted DNA were measured with a NanoDrop nd-1000 spectrophotometer (Thermo Fisher Scientific, Waltham, MA, USA) and through agglutinin gel electrophoresis, respectively. A microbial diversity library was constructed using universal primers (Additional file 10: Table S5). The construction and sequencing of the diversity library were performed using the Illumina MiSeq platform (PE300, CA, USA) from Personalbio (Shanghai, China). In this study, eight bacterial diversity libraries and eight fungal diversity libraries were constructed from two groups of samples. Each group had four replicates with 30 leaves each. For raw sequencing data, the representative OTU classification of each sample was obtained using the QIIME (v1.8.0) and R package (v3.2.0) [67]. All microbial diversity sequencing raw data were stored in the NCBI sequence read archive (SRA accession number: bacterial, PRJNA598468; fungal, PRJNA598022).

### Statistical analysis of data

Data were analyzed by Excel and SAS for ANOVA, and then SAS (SAS Institute Inc., Cary, NC, USA) was used for Duncan test (*p* ≤ 0.05) for significant difference.

## Supplementary Information

**Additional file 1: Figure S1. **Cultivar registration certificate of ‘Maguxianzi’.

**Additional file 2: Table S1. **Quality inspectionof sample sequencing data.

**Additional file 3: Table S2. **Evalution of sample alignment rate.

**Additional file 4: Table S3. **Detectionof sample alignment area.

**Additional file 5: Figure S2. **Correlation coefficient between FPKM of genes of samples.

**Additional file 6: Figure S3. **The number of total DEGs in M vs H. Compared with H, the DEGs up-regulated in M were represented by yellow, whereas the cyan represented the down-regulated DEGs in M.

**Additional file 7: Figure S4. **Clustering expression heatmap of DEGs in different samples. Different samples are marked in black front at the bottom of the figure. The expression level of genes in different samples indicated by a change of color, and the color change from blue to yellow suggest an increase of expression level of genes.

**Additional file 8: Figure S5. **The expression pattern clustering of DEGs in different samples. All DEGs were divided into 6 subclasses.

**Additional file 9: Table S4. **Primers used in qRT-PCR.

**Additional file 10: Table S5. **Universal primer used in microbial diversity sequencing.

## Data Availability

All raw data reported in this paper have been deposited in the National Center for Biotechnology Information sequence read archive (RNA-sequcing accession SRA accession number: PRJNA577010; Microbial diversity sequencing SRA accession number: bacterial, PRJNA598468; fungal, PRJNA598022). The database generated and the materials used during the current study are available from the corresponding and first authors on reasonable request (xufeng198@126.com; fumingyue1214@163.com).
