# Supplementary material for: Comparative transcriptome and microbial community sequencing provide insight into yellow-leaf phenotype of Camellia japonica
Source: BMC Plant Biol. 2021 Sep 10;21:416. doi: 10.1186/s12870-021-03198-w (PMC8431858; doi:10.1186/s12870-021-03198-w)
Supplement: Supplementary file 1 — Additional file 1: Figure S1. Cultivar registration certificate of ‘Maguxianzi’. [file 12870_2021_3198_MOESM1_ESM.pdf]

New Cultivar Certification of *Camellia japonica*

Registered Number: 2013 No.116

This certification is hereby awarded to the following varieties which meet the relevant standards for new *Camellia japonica* varieties after the appraisal of relevant experts organized by Chinese Camellia Registration Committee.

Chinese Name:	"Maguxianzi"
Scientific Name:	*Camellia japonica* cultivar "Maguxianzi"
Registrant:	Wunao Mountain National Forest Park of Macheng
Registered Adress:	Wunao Mountain National Forest Park of Macheng
Registered Date:	March 18, 2013

中国茶花命名和统一登录委员会

CCR
