# Supplementary material for: Comparative transcriptome and microbial community sequencing provide insight into yellow-leaf phenotype of Camellia japonica
Source: BMC Plant Biol. 2021 Sep 10;21:416. doi: 10.1186/s12870-021-03198-w (PMC8431858; doi:10.1186/s12870-021-03198-w)
Supplement: Supplementary file 2 — Additional file 2: Table S1. Quality inspectionof sample sequencing data. [file 12870_2021_3198_MOESM2_ESM.docx]

**Table S1. Quality inspection of sample sequencing data.**

| Sample | Raw reads | raw bases | clean reads | clean bases | clean reads rate (%) | Low-quality Reads Number | Low-quality Reads Rate (%) | Ns Reads Number | Ns Reads Rate (%) | Adapter Polluted Reads Number | Adapter Polluted Reads Rate (%) | Raw Q30 Bases Rate (%) | Clean Q30 Bases Rate (%) | GC Percent (%) |
| --- | --- | --- | --- | --- | --- | --- | --- | --- | --- | --- | --- | --- | --- | --- |
| H1 | 46,889,448 | 7,033,417,200 | 45,487,274 | 6,823,091,100 | 97.01 | 480,978 | 1.03 | 36,058 | 0.08 | 885,138 | 1.89 | 93.96 | 94.5 | 45.85 |
| H2 | 49,216,220 | 7,382,433,000 | 47,043,620 | 7,056,543,000 | 95.59 | 472,592 | 0.96 | 36,212 | 0.07 | 1,663,796 | 3.38 | 93.95 | 94.54 | 47.42 |
| H3 | 49,742,382 | 7,461,357,300 | 47,677,334 | 7,151,600,100 | 95.85 | 421,128 | 0.85 | 5,242 | 0.01 | 1,638,678 | 3.29 | 94.49 | 95.2 | 47.93 |
| M1 | 46,192,182 | 6,928,827,300 | 44,480,470 | 6,672,070,500 | 96.29 | 376,546 | 0.81 | 4,884 | 0.01 | 1,330,282 | 2.88 | 94.75 | 95.26 | 45.44 |
| M2 | 47,484,066 | 7,122,609,900 | 44,919,528 | 6,737,929,200 | 94.6 | 703,206 | 1.48 | 34,908 | 0.07 | 1,826,424 | 3.85 | 93.35 | 94.13 | 45.68 |
| M3 | 46,962,308 | 7,044,346,200 | 44,680,796 | 6,702,119,400 | 95.14 | 437,164 | 0.93 | 33,938 | 0.07 | 1,810,410 | 3.85 | 94.2 | 94.66 | 46.06 |
| Summary | 286,486,606 | 42,972,990,900 | 274,289,022 | 41,143,353,300 |  |  |  |  |  |  |  |  |  |  |
