# Supplementary material for: Comparative transcriptome and microbial community sequencing provide insight into yellow-leaf phenotype of Camellia japonica
Source: BMC Plant Biol. 2021 Sep 10;21:416. doi: 10.1186/s12870-021-03198-w (PMC8431858; doi:10.1186/s12870-021-03198-w)
Supplement: Supplementary file 3 — Additional file 3: Table S2. Evalution of sample alignment rate. [file 12870_2021_3198_MOESM3_ESM.docx]

**Table S2. Evaluation of sample alignment rate.**

| Sample | Total Reads | Mapped Reads | Mapping Rate (%) | UnMapped Reads | MultiMap Reads | MultiMap Rate (%) |
| --- | --- | --- | --- | --- | --- | --- |
| H1 | 45,487,274 | 30,731,475 | 67.56 | 14,755,799 | 1,610,717 | 3.54 |
| H2 | 47,043,620 | 31,036,021 | 65.97 | 16,007,599 | 2,646,774 | 5.63 |
| H3 | 47,677,334 | 31,223,102 | 65.49 | 16,454,232 | 3,021,205 | 6.34 |
| M1 | 44,480,470 | 30,251,419 | 68.01 | 14,229,051 | 1,474,344 | 3.31 |
| M2 | 44,919,528 | 30,764,699 | 68.49 | 14,154,829 | 1,494,422 | 3.33 |
| M3 | 44,680,796 | 30,938,029 | 69.24 | 13,742,767 | 1,573,085 | 3.52 |
