# Supplementary material for: Comparative transcriptome and microbial community sequencing provide insight into yellow-leaf phenotype of Camellia japonica
Source: BMC Plant Biol. 2021 Sep 10;21:416. doi: 10.1186/s12870-021-03198-w (PMC8431858; doi:10.1186/s12870-021-03198-w)
Supplement: Supplementary file 4 — Additional file 4: Table S3. Detectionof sample alignment area. [file 12870_2021_3198_MOESM4_ESM.docx]

**Table S3. Detection of sample alignment area.**

| Sample | Exon | Intron | Intergenic |
| --- | --- | --- | --- |
| H1 | 11,219,210(62.96%) | 615,881(3.46%) | 5,984,019(33.58%) |
| H2 | 10,172,144(56.91%) | 426,795(2.39%) | 7,275,998(40.71%) |
| H3 | 9,542,545(53.24%) | 358,150(2.00%) | 8,023,256(44.76%) |
| M1 | 10,825,024(61.53%) | 672,886(3.82%) | 6,093,909(34.64%) |
| M2 | 11,235,211(62.98%) | 653,592(3.66%) | 5,950,555(33.36%) |
| M3 | 11,261,499(63.15%) | 482,748(2.71%) | 6,090,090(34.15%) |
