# Supplementary figures and images for: Comparative transcriptome and microbial community sequencing provide insight into yellow-leaf phenotype of Camellia japonica
Source: BMC Plant Biol. 2021 Sep 10;21:416. doi: 10.1186/s12870-021-03198-w (PMC8431858; doi:10.1186/s12870-021-03198-w)

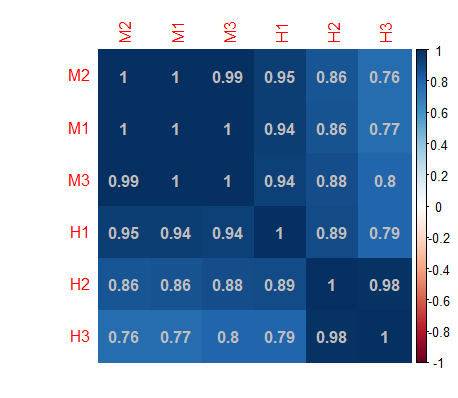

Supplement: Supplementary file 5 — Additional file 5: Figure S2. Correlation coefficient between FPKM of genes of samples. [file 12870_2021_3198_MOESM5_ESM.docx]

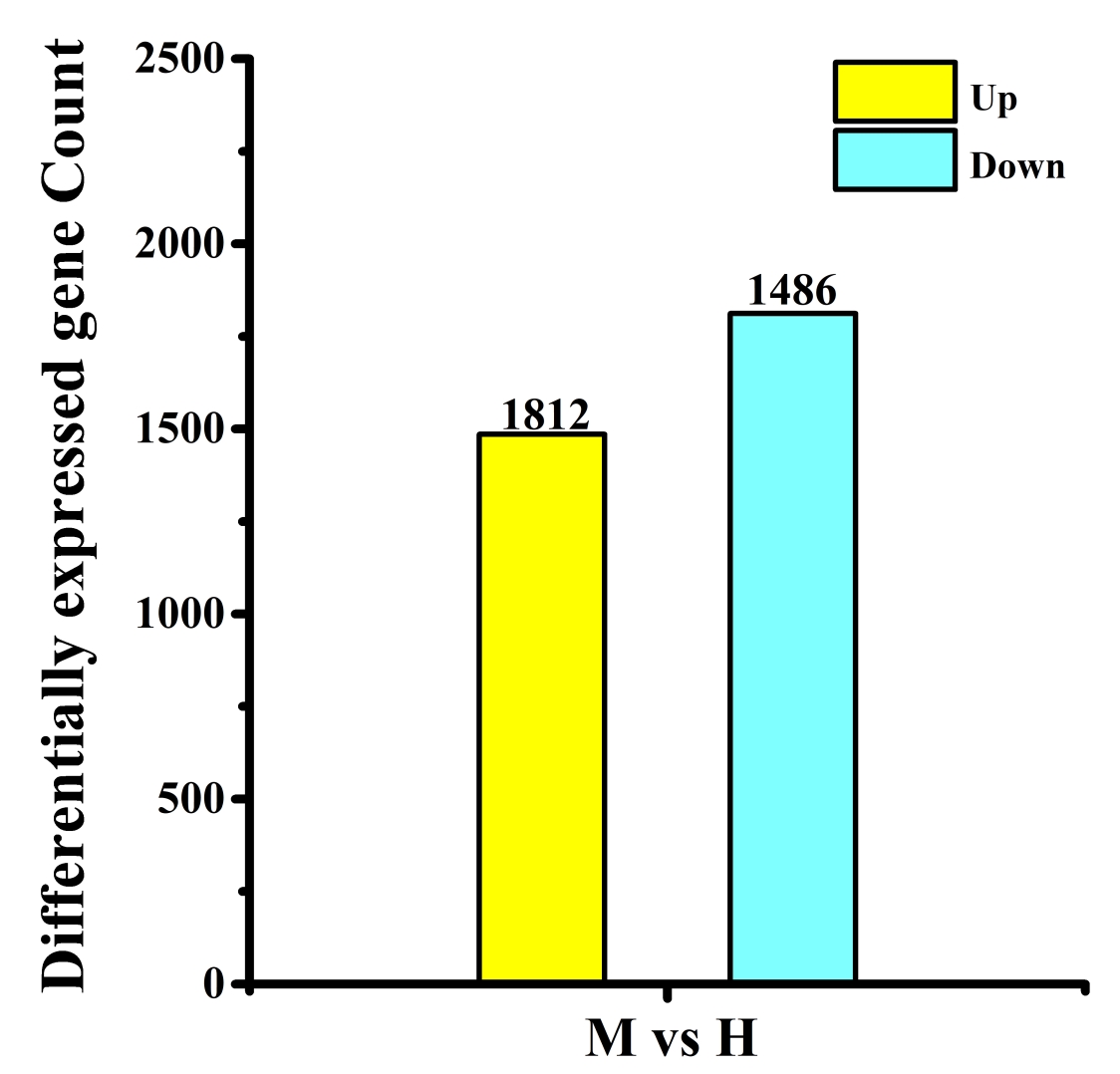

Supplement: Supplementary file 6 — Additional file 6: Figure S3. The number of total DEGs in M vs H. Compared with H, the DEGs up-regulated in M were represented by yellow, whereas the cyan represented the down-regulated DEGs in M. [file 12870_2021_3198_MOESM6_ESM.docx]

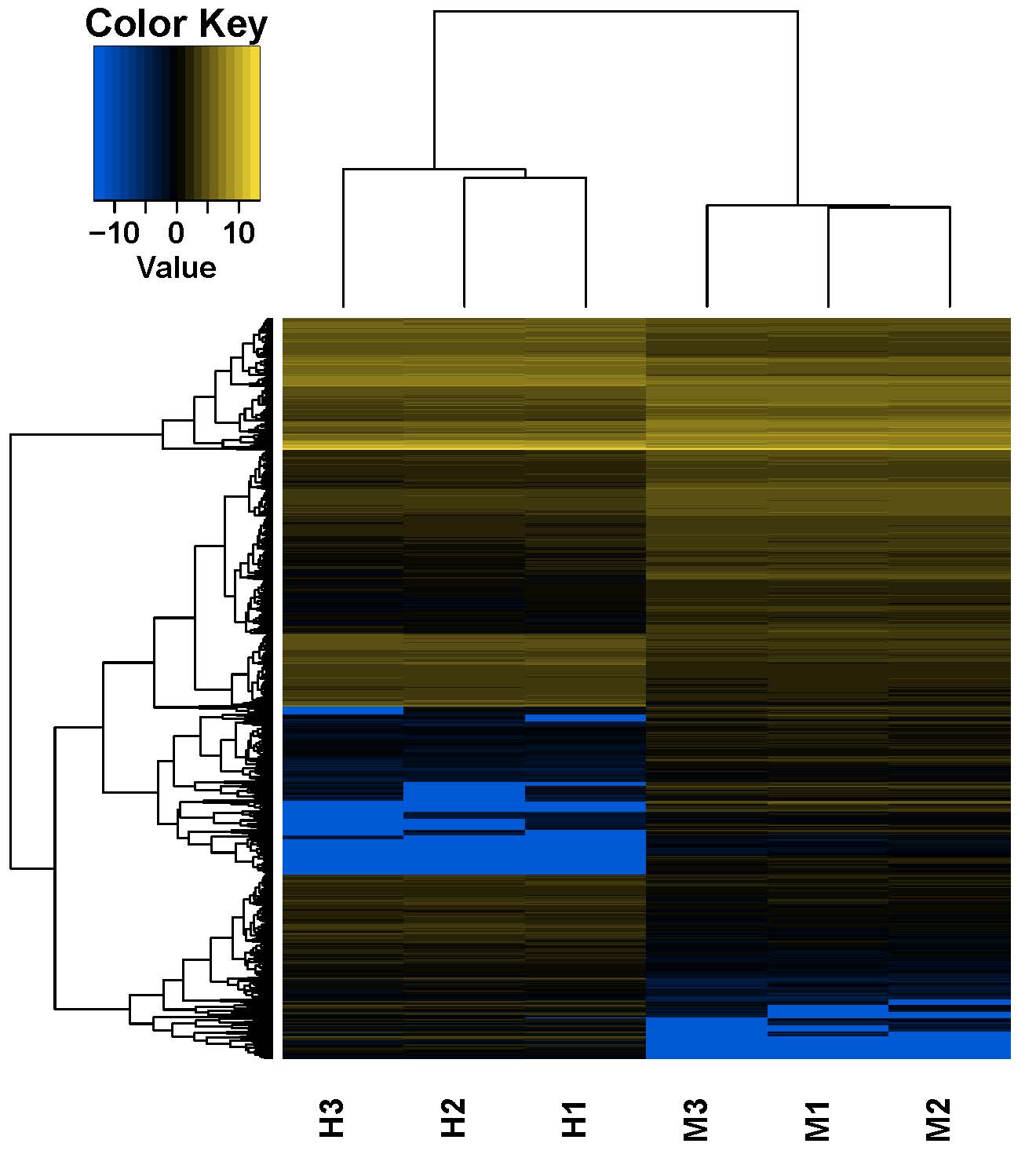

Supplement: Supplementary file 7 — Additional file 7: Figure S4. Clustering expression heatmap of DEGs in different samples. Different samples are marked in black front at the bottom of the figure. The expression level of genes in different samples indicated by a change of color, and the color change from blue to yellow suggest an increase of expression level of genes. [file 12870_2021_3198_MOESM7_ESM.docx]

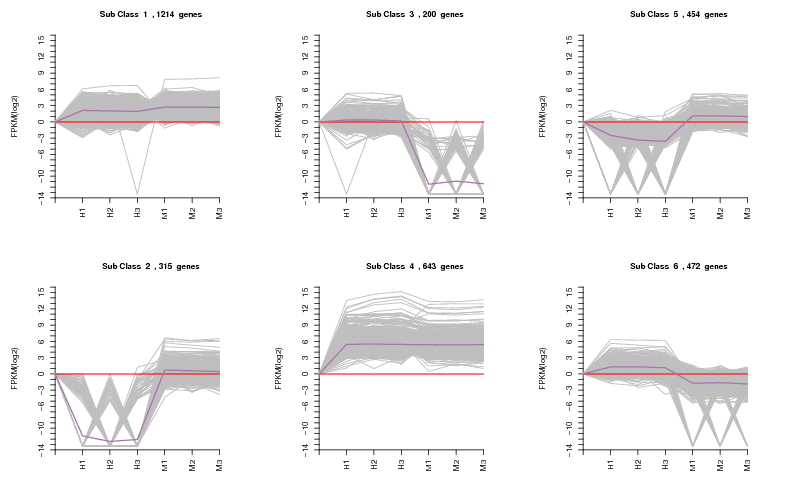

Supplement: Supplementary file 8 — Additional file 8: Figure S5. The expression pattern clustering of DEGs in different samples. All DEGs were divided into 6 subclasses. [file 12870_2021_3198_MOESM8_ESM.docx]
