# Supplementary material for: Comparative transcriptome and microbial community sequencing provide insight into yellow-leaf phenotype of Camellia japonica
Source: BMC Plant Biol. 2021 Sep 10;21:416. doi: 10.1186/s12870-021-03198-w (PMC8431858; doi:10.1186/s12870-021-03198-w)
Supplement: Supplementary file 9 — Additional file 9: Table S4. Primers used in qRT-PCR. [file 12870_2021_3198_MOESM9_ESM.docx]

**Table S4. Primers used in qRT-PCR.**

| **Gene** | **Primer** | **Forward and reverse primers (5’- 3’)** | **ID** | **Annotation** |
| --- | --- | --- | --- | --- |
| glutamyl-tRNA reductase (HemA) | Sense | GTGGCTTCGCTACCTCTATTG | CSA009845 | Porphyrin and chlorophyll metabolism |
|  | Anti-sense | TATGGCCTTCGACGGTTTATG |  |  |
| heme oxygenase (HMOX1) | Sense | CCCAATTTCACTGGAAAGTCTTC | CSA030148 | Porphyrin and chlorophyll metabolism |
|  | Anti-sense | CATGGCCACAAACCTCATTTC |  |  |
| Chlorophyllase (CLH) | Sense | GGGACTGAGGAAGTTGATTCTG | CSA027244 | Porphyrin and chlorophyll metabolism |
|  | Anti-sense | AGGGCTAGTTTGAGGAGATTTG |  |  |
| zeta-carotene isomerase (Z-ISO) | Sense | GCCATGAGGTTGTGATGTTTAC | CSA033962 | Carotenoid biosynthesis |
|  | Anti-sense | GCACGTTCTCCAATGAGTTTC |  |  |
| beta-ring hydroxylase (CYP450-BCH) | Sense | GTGGGAGTAGCACCAATAGAAG | CSA034253 | Carotenoid biosynthesis |
|  | Anti-sense | AGCATCGCATCTCTTCACTAC |  |  |
| carotene epsilon-monooxygenase (LUT1a) | Sense | GCACAAGAGGAAGTGGATAGAG | CSA023948 | Carotenoid biosynthesis |
|  | Anti-sense | AGGATGTGGGTAGAGACGAA |  |  |
| carotene epsilon-monooxygenase (LUT1b) | Sense | GCAGGAATACGGACCCATTTA | CSA023949 | Carotenoid biosynthesis |
|  | Anti-sense | TTCAGAGACCTCAGCAACAAG |  |  |
| zeaxanthin epoxidase (ZEP) | Sense | AGCTACACTTGTTACACTGGTATTG | CSA028595 | Carotenoid biosynthesis |
|  | Anti-sense | GCTTCGCTGTCAAGCCTTAT |  |  |
| 9-cis-epoxycarotenoid dioxygenase (NCED1) | Sense | CTTCGTAGCAAAGGACCCTAAC | CSA003404 | Carotenoid biosynthesis |
|  | Anti-sense | CTAGAAACCTCGATTCGCCTATG |  |  |
| 9-cis-epoxycarotenoid dioxygenase (NCED2) | Sense | CGGAGATGAGGTGGTTTGAA | CSA003405 | Carotenoid biosynthesis |
|  | Anti-sense | GTATGCTCCACCGCCAATA |  |  |
| 9-cis-epoxycarotenoid dioxygenase (NCED3) | Sense | CTTGAATTGGGCTCTCTCTCTAC | CSA024162 | Carotenoid biosynthesis |
|  | Anti-sense | CAGGAGTTGGAGTTGGTGTT |  |  |
| (+)-abscisic acid 8'-hydroxylase (CYP707A) | Sense | TTGTGCCAAAGCCCTATACA | CSA002949 | Carotenoid biosynthesis |
|  | Anti-sense | GACTAGGTGGTGGGTCAATATG |  |  |
| abscisate beta-glucosyltransferase (AOG1) | Sense | TTTGGGTGGCCAGAGATATG | CSA022206 | Carotenoid biosynthesis |
|  | Anti-sense | CATCCTCTCCTCAAATCCATCC |  |  |
| bifunctional dihydroflavonol 4-reductase/flavanone 4-reductase (DFR) | Sense | TCGGTTCATGGCTCGTTATG | CSA035727 | Flavonoid biosynthesis |
|  | Anti-sense | TTGGGCAAGTCCAAGAGATG |  |  |
| flavonol synthase (FLS) | Sense | CTACCACTTCTGGCCTAAGAAC | CSA008358 | Flavonoid biosynthesis |
|  | Anti-sense | CCCAATCCCTAATGACAACCA |  |  |
| caffeoyl-CoA O-methyltransferase (CCoAOMT) | Sense | CTCCTGATGCAGGTCAGTTAAT | CSA001731 | Flavonoid biosynthesis |
|  | Anti-sense | GGAAAGAGCAGTGAGGAGAAG |  |  |
| photosystem II PsbY protein (psbY) | Sense | CTCCAACCAGCTCTCAATCAA | CSA036170 | Photosynthesis |
|  | Anti-sense | GCTGAAGCATGAGGAGTCATAG |  |  |
| photosystem I subunit PsaO (psaO) | Sense | CGATCAACGGAAACAGCTTAAC | CSA031421 | Photosynthesis |
|  | Anti-sense | ACCTCAACACAACAGAATGGA |  |  |
| light-harvesting complex I chlorophyll a/b binding protein 2 (LHCA2) | Sense | GTGAACCACTTCCTCCTGATAG | CSA020482 | Photosynthesis - antenna proteins |
|  | Anti-sense | CCCTAATCCAAGTGGGTCAAA |  |  |
| light-harvesting complex II chlorophyll a/b binding protein 3 (LHCB3） | Sense | TCCAAGCTATTGTCACTGGTAAA | CSA016997 | Photosynthesis - antenna proteins |
|  | Anti-sense | GGTACAAACTTGGTGGCATAGA |  |  |
