# Supplementary material for: Comparative transcriptome and microbial community sequencing provide insight into yellow-leaf phenotype of Camellia japonica
Source: BMC Plant Biol. 2021 Sep 10;21:416. doi: 10.1186/s12870-021-03198-w (PMC8431858; doi:10.1186/s12870-021-03198-w)
Supplement: Supplementary file 10 — Additional file 10: Table S5. Universal primer used in microbial diversity sequencing. [file 12870_2021_3198_MOESM10_ESM.docx]

**Table S5.** **Universal primer used in microbial diversity sequencing.**

| Primer | Sequence of primer (5’- 3’) |
| --- | --- |
| 338F | ACTCCTACGGGAGGCAGCA |
| 806R | GGACTACHVGGGTWTCTAAT |
| ITS5 | GGAAGTAAAAGTGGTAACAAGG |
| ITS1 | GCTGCGTTCTTCATCGATGC |
